# Membrane Lipid Composition: Effect on Membrane and Organelle Structure, Function and Compartmentalization and Therapeutic Avenues

**DOI:** 10.3390/ijms20092167

**Published:** 2019-05-01

**Authors:** Doralicia Casares, Pablo V. Escribá, Catalina Ana Rosselló

**Affiliations:** 1 Laboratory of Molecular Cell Biomedicine, University of the Balearic Islands, 07121 Palma de Mallorca, Spain; doralicia.casares@lipopharma.com (D.C.); pablo.escriba@uib.es (P.V.E.); 2 Lipopharma Therapeutics, Isaac Newton, 07121 Palma de Mallorca, Spain

**Keywords:** membrane, lipid, therapy, endomembrane, structure, composition, 2OHOA

## Abstract

Biological membranes are key elements for the maintenance of cell architecture and physiology. Beyond a pure barrier separating the inner space of the cell from the outer, the plasma membrane is a scaffold and player in cell-to-cell communication and the initiation of intracellular signals among other functions. Critical to this function is the plasma membrane compartmentalization in lipid microdomains that control the localization and productive interactions of proteins involved in cell signal propagation. In addition, cells are divided into compartments limited by other membranes whose integrity and homeostasis are finely controlled, and which determine the identity and function of the different organelles. Here, we review current knowledge on membrane lipid composition in the plasma membrane and endomembrane compartments, emphasizing its role in sustaining organelle structure and function. The correct composition and structure of cell membranes define key pathophysiological aspects of cells. Therefore, we explore the therapeutic potential of manipulating membrane lipid composition with approaches like membrane lipid therapy, aiming to normalize cell functions through the modification of membrane lipid bilayers.

## 1. Introduction

Biological membranes define cell boundaries and internal organelles in eukaryotes. These assemblies are highly dynamic in order to allow maintenance of the integrity and identity of the enclosed structures [1]. In 1972, the publication by Singer and Nicolson of the fluid mosaic model of the structure of cell membranes [2] encouraged the study of membranes and the role of each of their components. Membranes are formed by a fluid lipid bilayer which confers exceptional physical properties to the cell [3] and whose lipids interact with proteins by hydrophobic and Coulomb forces [4]. These interactions allow membranes to create a variety of domains based on the type of lipid components [5]. Those domains, in turn, conform different structures and exert specific functions, such as the propagation of different cell signals [6]. To maintain their structure, membranes also interact with cytoskeleton [7].

Membranes are essential for cell survival and can provide us with information about the origin of life and other events in cell history (e.g., bioenergetic organelles acquisition or endomembrane system formation) [1]. However, the word evolution is not usually related to biomembranes in textbooks [4], and the origins and evolution of the membrane trafficking system have not been properly addressed [8]. To study the evolution of membranes, the nature optimization of biological properties has to be taken into account [4]. For many years, different types of cells were distinguished by the presence (eukaryotes) or absence (prokaryotes) of a nucleus. We now know that prokaryotes can also be separated into bacteria and archaea which share many characteristics and come from a common ancestor called LUCA (last universal common ancestor) [1]. One differential trait between archaea and bacteria is their different lipid membrane composition due to differences in the enzymes that synthesize them [1]. Eukaryotes, which were formed later in life history, have a similar lipid biochemistry to bacteria, but not to archaea (reviewed in [1]).

The first cellular systems on Earth arose from three molecular species: molecules which stored information for replication, catalysts encoded by that information and molecules which could encapsulate both previous species [9]. Also, these primitive cells needed energy-storing molecules to create ordered biologically active molecules [10]. In fact, as reviewed by Gould, ATP synthase is conserved in bacteria and archaea while membrane lipids are not, which points to the fact that lipid biosynthesis was a late step in the emergence of cells, but an essential trait to acquire a free-living state [1]. Although we can find prokaryotes with internal compartments, none of them have developed the endomembrane system eukaryotes possess [1,8] and these structures are not homologous to eukaryotic structures [11]. However, all three domains of life (Bacteria, Archaea and Eukarya) have replication machinery, transcription, translation and key metabolic pathways, which suggest that these systems must have been already present in proto-eukaryotes [12].

Eukaryogenesis increased cell complexity by creating new membranous compartments which carry out specialized functions and vesicle trafficking with a specific source and destination [1]. The development of this trafficking system, as reviewed in [8], has been explained by two different hypotheses over the years: endosymbiotic theory and autogenesis. In modern studies, eukaryotes derive from the integration of proteobacterium (now mitochondria) into archaeal cells, however, the complexity of the archaeal ancestor at that time is still subject to debate [1]. Evidence such as the similarity between mitochondria-derived vesicles and prokaryotes’ outer membrane vesicles support this theory [1]. Another controversial point is the speed of this trafficking-system evolution. The last eukaryotic common ancestor (LECA) did probably possess an internal complexity so the evolution might have been fast [8]. According to the presence of different organelles, the LECA is told to have possessed a basic membrane-trafficking system including an endoplasmic reticulum, stacked Golgi, endosomes and lysosomes, which are now shared by all studied eukaryotes [8]. In fact, there is a common core of protein factors involved in transport and compartment specificity [13]. Genomic studies further demonstrate these LECA features studying the members of the coat protein complex I and II (COPI, COPII) and clathrin complexes, and on the soluble N-ethylmaleimide-sensitive factor (NSF) attachment protein receptor (SNARE) families, Guanosine-5’-triphosphate hydrolases (GTPase)–activating proteins, guanine nucleotide exchange factors (GEFs) and NSF ATPases, endosomal sorting complexes required for transport (ESCRT) complexes, GTPases and homologues of the retromer complex (reviewed in [8]). Different organelles share homologous membrane-trafficking components originated from gene duplication which might have occurred simultaneously to organelle formation or might even be itself responsible for organelle development [8]. In addition, other trafficking components were apparently added separately while some others were lost throughout membrane evolution in the adaptation of LECA’s descendants to new environments [8].

Four billion years of evolution that began with prokaryotic cells produced the current eukaryotic cells containing different organelles with a particular membrane composition and function. Membrane lipid composition in the plasma membrane (PM) and endomembrane compartments is responsible of sustaining organelle structure and function and is reviewed here. Studying the diversity of lipid species in membranes has become a field of research in itself. Eukaryotic cell membranes are formed by hundreds of lipid species assembled following a functional asymmetry [1]. What is more, alterations in membrane lipid composition generate pathological conditions in cells and manipulating the membrane lipid composition is a therapeutic approach beginning to be exploited with strategies like membrane lipid therapy.

## 2. Repertoire of Membrane Lipids

Membrane lipids are the least studied biomolecules. First, the tools for the study of lipids are not as powerful as the tools used for investigating genes and proteins. Second, lipids occur in membranes in the form of ensembles, including around tens of thousands of individual members, which in turn are representative of a variety of hundreds of different molecular species [14]. Third, the high plasticity and flexibility of biological membranes allow them to maintain their bioactivity despite the effect of external insults (reviewed in [15]). Nonetheless, cells depend upon lipids for three main functions, namely energy storage, compartmentalization and signaling (reviewed in [16]):

1. Energy storage: lipid droplets used for this function contain mainly triacylglycerol and steryl esters thanks to their relatively reduced state. These anhydrous reservoirs are needed for the efficient storage of caloric reserves and as stores of fatty acid and sterol components for membrane biogenesis.
2. Compartmentalization: the milieu of cellular membranes is made of lipids of amphipathic nature, comprising both a hydrophobic and a hydrophilic portion. This amphipathic nature provides the physical basis for spontaneous membrane formation because the hydrophobic moieties are prone to self-associate when dissolved in water. This predisposition to self-associate enabled the segregation of an internal milieu from the external milieu when the first cells originated. Later on, this scheme was repeated inside the cell to generate discrete organelles allowing, first, the separation of specific chemical reactions, second, the limitation in the spreading of reaction products and, third, an improvement in biochemical efficiency. Furthermore, lipids are responsible of membrane ability of budding, tubulation, fission and fusion, all them indispensable for cell division, biological reproduction and intracellular membrane trafficking.
3. Signaling: in signal transduction, lipids first define membrane domains that allow the aggregation and dispersion of particular proteins, and subsequently organize secondary signaling or effector complexes; they can also act as first and second messengers. The rupture of amphipathic lipids generates bipartite signaling elements, which can be spread both within a membrane (by hydrophobic portions of the molecule) and through the cytosol (by soluble/polar portions of the molecule).

**Figure 1:**
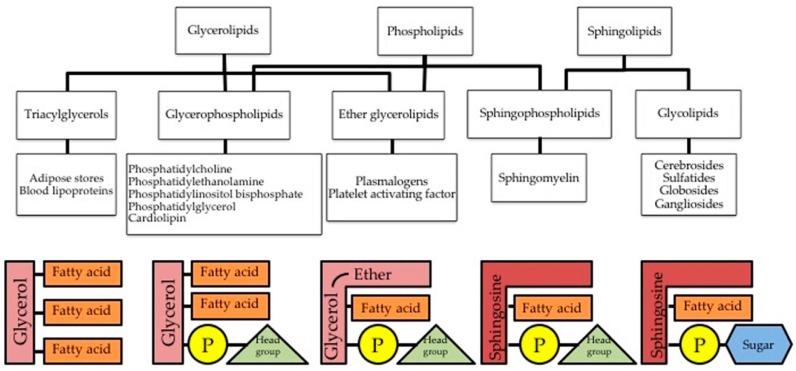
Several forms of triglycerides and phospholipids found in the cell. The left-most scheme is a triglyceride formed by a three-carbon glycerol backbone with a FA tail bound to each carbon. Second left-most scheme is a phospholipid bearing two FA tails with the third carbon of glycerol bound to a phosphate group. The middle scheme is an ether glycerolipid, who shares with the two previous structures the three-carbon glycerol backbone and is slightly modified with an ether group. In second right-most scheme, a sphingosine is shown as backbone instead of glycerol and first fatty acid tail of glycerol is modified slightly. This is a sphingophospholipid since it bears a phosphate group, it is called sphingomyelin. Right-most scheme shows the scheme of sphingolipids called glycolipids, since the phosphate group is replaced with a sugar (carbohydrate group).

Phospholipids (PL) constitute the bulk of the membrane’s lipid matrix (Figure 1). They resemble the triglycerides in being ester or amide derivatives of glycerol (glycerophospholipids, GPL) or sphingosine (Sph –sphingolipids, SL) with fatty acids and phosphoric acid. 

Glycerophospholipids (GPL) are the major structural lipids in eukaryotic membranes. They are generally composed of two fatty acids linked through two hydrophobic acyl chains and a phosphate head group ester linked to a glycerol. In GPL, the phosphate moiety of the resulting phosphatidic acid is further esterified with choline, ethanolamine, serine or inositol in the phospholipid itself. Therefore, GPL branch from phosphatidic acid (PA) and are classified upon the structure of the PL head group. Phosphatidylcholine (PtdCho) and phosphatidylethanolamine (PtdEtn) heads are zwitterionic, whereas phosphatidylserine (PtdSer) and phosphatidylinositol (PtdIns) heads are anionic (reviewed in [16,17]). Each of these phospholipid classes is defined by a shared structure but then includes a battery of molecular species upon the length and degree of saturation in their acyl chains. Both head group and acyl chain composition influence the physical properties of the membrane [18]. 

Sphingolipids (SL) are the second most abundant structural lipid. SL contain one hydrophobic acyl chains and a phosphate head group ester linked to a Sph backbone. Their hydrophobic backbone is an ester or amide derivative of Sph with fatty acids being ceramide (Cer) the simplest representative. Sphingomyelin (SM) contains a phosphorylcholine headgroup associated to the sphingoid base. SM is the more abundant SL in the plasma membrane (PM) of mammalian cells. Within the total PL fraction of the PM, SM accounts for 2%–15% upon the cell type (reviewed in [19]). Other SLs are glycosphingolipids (GSLs). GSLs are based on glucosylceramide (GlcCer) or on galactosylceramide (GalCer) and contain mono-, di- or oligosaccharides [20].

GPL and SL generate comparable series of messenger lipids upon signaling-induced hydrolysis. GPL hydrolysis produces the messenger lipids: lysoPtdCho (LPC), lysoPA (LPA), PA and diacylglycerol (DAG). Whereas SL hydrolysis produces the messenger lipids: sphingosylphosphorylcholine (SPC), Sph, sphingosine-1-phosphate (S1P), ceramide-1-phosphate (C1P) and Cer (reviewed in [16]).

Cholesterol (Chol) is a crucial element of mammalian cell membranes. Chol molecule involves four fused rings (steroid backbone) showing a hydroxyl group (A-ring) and a small branched hydrophobic tail (D-ring). In membranes, the rigid steroid backbone of Chol favors its interaction with SL. Chol-SL platforms are the basic element of lipid rafts. Chol content is strictly controlled by three mechanisms: de novo synthesis, uptake and esterification [21].

## 3. Lipids in Sustaining Organelle Structure, Function and Identity

**Figure 2:**
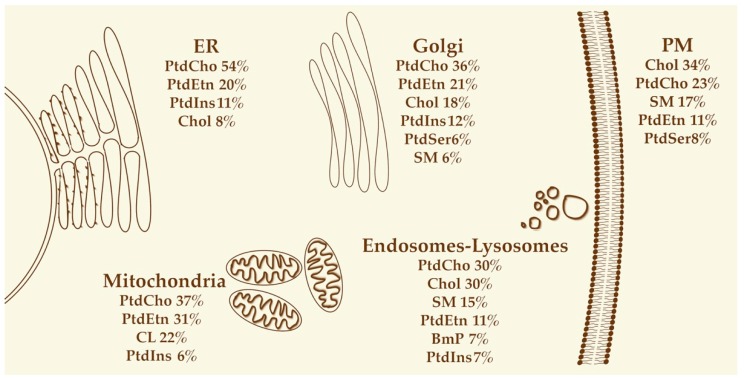
Lipids in organelle identity. For each organelle, the main lipid components are listed according to their contribution to the total lipid content of the organelle (in percentage, from the most abundant to the least abundant). Only lipids representing at least 5% of the total lipids for each organelle are shown. BmP: bis(monoacylglycero)phosphate; Chol: cholesterol; CL: cardiolipin; PtdCho: phosphatidylcholine; PtdEtn: phosphatidylethanolamine; PtdIns: phosphatidylinositol; PtdSer: phosphatidylserine; SM: sphingomyelin. Not to scale. Adapted from [19].

Lipids are distributed heterogeneously in several ranges: subcellular organelles show varied lipid arrangements, furthermore PM and organelle membranes present foci of specific lipid domains, and finally lipid distribution shows lateral differences and/or transversal asymmetry, i.e., asymmetric distribution in both membrane leaflets (Figure 2, reviewed in [22]).

### 3.1. Endoplasmic Reticulum

The secretory pathway is the best characterized model of lipid localization and involves the endoplasmic reticulum (ER), Golgi apparatus, and PM. These three compartments display growing amounts of Chol and SL concentrations, with higher levels in PM. In fact, this Chol and SL gradient is responsible of the increased membrane thickness and stiffness of the PM. These two physical properties (thickness and stiffness) are crucial to determine the particular functions and identities of the different organelles. Furthermore, they might be related with the sorting of membrane proteins and lipids between the organelles. Because of this, lipid composition and, further on, membrane thickness and stiffness, must be homeostatically maintained [23,24,25,26]. The ER, for instance, maintains its membrane fluidity using sensory machinery [27,28], which also operates to pair the synthesis of proteins and lipids with membrane biogenesis needs [29,30]. 

The ER produces the bulk of the structural PL and Chol, together with significant levels of triacylglycerol and cholesteryl esters with non-structural roles [31]. In addition, the ER produces Cer, which is the precursor for complex SL. In the ER of myelinating and epithelial cells GalCer is produced to stabilize myelin and apical membranes [32]. Chol in the ER is only about 1%–2% of total Chol because, right after being synthesized, Chol is transferred from the ER to the PM. This ATP-dependent and non-vesicular transport is extremely rapid: in mammals, the half-life of Chol in the ER is only 10–20 min (reviewed in [22]). 

The fast transport of Chol and other lipids to other organelles causes a loose arrangement of membrane lipids in ER membrane. This loose lipid organization is critical for ER function as it eases the insertion and the transport of recently synthesized lipids and proteins (reviewed in [16]). Furthermore, the ER is the main supplier for a large percentage of membrane lipids in the Golgi and PM, which are distal secretory organelles with restricted or null capacity to produce their own lipids (reviewed in [17]).

In addition, the ER includes minor lipids known to be both pathway intermediates and legitimate pathway end products (including DAG, cytidine diphosphoDAG, PA, lysophospholipids) and dolichol (reviewed in [16]).

Finally, a subfraction of the ER, that cofractionates with mitochondria and is known as the mitochondria-associated membrane, is particularly enriched in specific lipid biosynthetic enzymes. 

### 3.2. Golgi

Mammalian Golgi is the main producer of SL: SM, GlcCer, lactosylceramide (LacCer) and higher-order GSLs, whose final destination is the PM [33]. For SL production in the lumen of the trans-Golgi, Cer issued by the ER is required [34]. Later on, SL will accumulate in the PM whereas SL levels in the ER will be low. As it happens with SL, sterols are scarce in the ER, representing only 5 mol% of lipids, in contrast to the trans-Golgi and PM where sterols are abundant (30 mol%–40 mol%) [16,35]. Sterols favorably interact with lipids containing large head groups and saturated fatty acyl chains, as is the case for SL [36].

Both SL and sterols will eventually concentrate on the PM. This, together with a remodeling of PL acyl chains to a more saturated pattern, generates a tight lipid packing of sterols with PL. This packing is indeed responsible of the rigidity and thickness of this barrier isolating the intracellular from the extracellular milieu. The trans-Golgi is critical in the transition from the thin and loosely packed membrane found in the ER to the thick and rigid bilayer found at the PM. This transition, mediated by SL and sterols, is easily recognized following the single-spanning membrane proteins involved in the secretory pathway. Those membrane proteins contain transmembrane domains (TMDs) whose length and composition vary during the transition from the ER to the PM. Thus, for ER and Golgi-resident proteins, TMDs are considerably shorter than those for PM proteins [34]. Both TMD length and volume decide the fate of these proteins, i.e., Golgi retention or export to the PM [37,38]. Since selective associations between matching lipids and TMDs have proven sufficient to drive their co-segregation in model membranes [39], the preservation of the distinctive mixtures of membrane proteins and lipids that allow the ER and PM to execute their fundamentally unique tasks might be achieved by the Golgi exploiting physical principles such as hydrophobic mismatch and packing density. In turn, the outlined different TMD features in ER and PM exert temporal and spatial control over the main events governing the secretory pathway. Hence, early secretory organelles contain proteins bearing neutral amphipathic lipid packing sensor (ALPS) motifs. Those proteins show bulky hydrophobic residues that easily accommodate into lipid-packing imperfections. In contrast, late secretory organelles concentrate peripheral membrane proteins bearing polybasic motifs [40,41,42,43].

### 3.3. Plasma Membrane

Different cell types contain different lipid and protein compositions of their PM. Furthermore, PM is compartmentalized into microdomains with specific physico-chemical properties, e.g., signalosomes, the platforms where signaling partners exert productive interactions. Those microdomains are defined by the specific type and abundance of a given collection of proteins and lipids, including fatty acid specific composition of the latter. As an example, the cis-monounsaturated FA, oleic acid, leads the arrangement of nonlamellar phases in model membranes, in contrast to its saturated and trans-monounsaturated counterparts (stearic and elaidic, respectively) [44]. Indeed, varied raft microdomains exist upon their specific lipid composition and subsequent biophysical properties [45]. Lipid rafts are rich in SM, GSLs and Chol which arrange into rigid and liquid-ordered (Lo) microdomains. In contrast, the small polar heads of PtdEtn endow PtdEtn-rich regions with more fluidity and less surface packing density and pressure, these regions are then called liquid-disordered (Ld) microdomains [46]. Lipid heterogeneous distribution gives rise to both transient and stable membrane microdomains. Transient membrane microdomains include lipid rafts, caveolae and coated pits, among others, while stable membrane microdomains involve tight junctions, synaptosomes, brush border, etc. This segregation between transient and stable membrane microdomains is led by specific proteins and remarkably by lipids such as Chol [45,47,48]. 

Furthermore, in eukaryotes, both leaflets of the PM contain specific lipid compositions. The outer leaflet of the PM contains mostly PtdCho and SLs. The inner leaflet, in contrast, involves PtdEtn, the negatively charged PtdSer, and PtdIns [49]. This membrane asymmetry is created and maintained by two types of ATP-driven pumps: flippases and floppases [50,51,52]. A rapid collapse of these pumps, called phospholipid scrambling, externalizes PtdSer allowing it to exert its effector role in many cell activities, for instance, the phagocytic clearance of apoptotic bodies and blood coagulation [53,54,55,56,57,58] (and reviewed in [59]).

PM does not participate in autonomous synthesis of its structural lipids, as above mentioned. However, in several signaling cascades, the synthesis or degradation of lipids at the PM has been extensively reported [60]. For SM turnover, for example, the PM contains sphingomyelin synthase 2 (SMS2), which allows the (re)synthesis of SM from Cer at PM and even has a partial effect on whole cell SM content [61,62].

The multiple functions of membranes depend on this lipid composition, which confers relevant functions to cells. Signalosomes, by instance, may mediate incomplete signaling cascades whose activation depends upon the recruitment of one further signaling entity (e.g., the receptor) to the lipid raft [63]. 

#### Endosomes

PM lipid domains are indispensable for the assembly of signalosomes and subsequently play a key role in their internalization [64,65]. Remarkably, the role of PL in internalization and maturation is biologically so significant among different phyla that it has been conserved during evolution. In all eukaryotic organisms and their compartments, PL work as membrane identifiers, controlling the circulation of individual compartments (reviewed in [66]). PL are not only bulk building units of membranes, but also operational controllers of main physiological events e.g., cytokinesis, chemotaxis and exocytosis. In addition, PL confer plasticity to biological membranes, for instance the PM continuously undergoes deformation, invagination, scission and fusion. Intracellular budding from the PM is the starting point of endocytosis (reviewed in [22]). Endocytosis comprehends several unrelated internalization routes, e.g., phagocytosis, clathrin-mediated endocytosis, and macropinocytosis. PM lipids are continuously endocytosed and later recycled in return to the PM (reviewed in [66]).

Together with the GPL, Chol and SL crucially participate in several routes of endocytosis, mainly the caveolae and the clathrin-independent carrier/glycosylphosphatidylinositol (GPtdIns)-anchor-enriched endocytic compartment-mediated (CLIC/GEEC) pathways (reviewed in [66]).

All along the endocytic pathway, the presence and abundance of specific lipids was found to be finely tuned. While early endosome lipid composition matches pretty much that of the PM, alongside maturation to late endosomes, sterols and PtdSer are decreased in favor of a massive rise in bis(monoacylglycero)phosphate (BmP) [67]. BmP is later implicated in fusion events, in production of multivesicular bodies, and in SL hydrolysis [68,69]. A dedicated collection of kinases and phosphatases generate and terminate specific phosphoinositides, among them phosphatidylinositol 4,5-bisphosphate (PtdIns(4,5)P2) on plasma membranes, phosphatidylinositol 3,4,5-trisphosphate (PtdIns(3,4,5)P3) on early endosomes, phosphatidylinositol 4,5-bisphosphate (PtdIns(3,5)P2) on late endosomes and phosphatidylinositol 4-phosphate (PtdIns4P) on the (trans)-Golgi network [70]. As already mentioned, the production of SL may have an important role in the sorting of membrane proteins and lipids between the ER, the plasma membrane and endosomes, or the vacuole through lipid rafts.

### 3.4. Mitochondria

Mitochondria rely on lipid and protein import for proper function [71]. Mitochondria present an intricate structure enclosing two sheaths: the outer (OMM) and the inner mitochondrial membrane (IMM). In turn, the intensely folded cristae generate two aqueous compartments, i.e., the intermembrane space (IMS) and the matrix (reviewed in [72]).

While mitochondria were shown to synthesize several lipids on their own, they pretty much rely upon the transference and gathering of lipids mostly produced in the ER (reviewed in [73,74,75,76,77,78,79,80]). Lipids autonomously synthesized by mitochondria include phosphatidylglycerol (PtdGro), cardiolipin (CL diphosphatidylglycerol) and in part PtdEtn, PA and cytidine diphosphate diacylglycerol (CDP-DAG). The major sites of lipid biosynthesis are the ER and its specific subfraction, the mitochondria associated membrane (MAM). Other mitochondrial membrane lipids such as PtdCho, PtdSer, PtdIns, sterols and SL have to be imported. The continuous supply and exchange of lipids is required for maintaining mitochondrial membrane integrity and overall cellular function (reviewed in [72]).

Mitochondrial lipid composition is mostly shared by all different mammalian cells and tissues and is characterized by: (i) Low PL to protein ratio and sterol to protein ratio versus other subcellular fractions. (ii) High levels of PtdCho and PtdEtn (major PL members near 80% of total PL); (iii) High CL content (10%–15% of total lipid composition), and (iv) Low sterols and SL amounts (reviewed in [72]). The bacterial origin of this membrane is revealed by the occurrence, in the IMM, of PtdGro and of CL (up to 25 mol%) and by the high PtdEtn/PtdCho ratio. In turn, those two features might be mandatory for oxidative phosphorylation [81]. Regarding the low sterol content of mitochondria, an exception is found in cells implicated in steroid hormone synthesis. The mitochondria of those cell types coordinate with the ER for the importing and metabolization of cholesterol [82]. Other exceptions are mitochondria from heart, brain and other tissues which additionally contain 5%–30% of total PL of PtdCho and PtdEtn plasmalogens [83,84]. Plasmalogens are a class of PL whose structure confers to them strong lipophilic properties. This feature of plasmalogens allows them to generate inverse hexagonal phase structures, which in turn promote membrane fusion [85,86]. Plasmalogens are the major membrane lipid in neurons [86,87,88,89] and, in specific tissues, they are crucial for the function of several transmembrane proteins and for membrane-related cholesterol transport both at the intracellular and extracellular levels [86,87,88,89].

Recent mass spectrometry-based lipidome analyses confirmed lipid species patterns described in mitochondria with earlier analysis techniques. Lipidome analyses fully recapitulate the found differences in FA composition of every lipid class, while this is only partially true for organelles. The role of mitochondrial lipids is now recognized for almost all lipid classes. Some of these lipids of mitochondrial membrane might be dispensable, but most contribute to the structural and functional characteristics of this organelle in a quite specific manner. Particularly mitochondrial lipids were shown to be a feasible target to modulate of the arrangement of mitochondrial supercomplexes (reviewed in [72]).

### 3.5. Lysosomes

Lysosomes contain the enzymatic tools for the degradation of extracellular molecules. The transcription factor EB (TFEB) partially controls lysosome biogenesis [90]. TFEB effect first enhances lysosomal exocytosis [91], second promotes the degradation of autophagic substrates and third helps the clearance of lipid droplets and of mitochondria [92,93,94]. Low amounts of cholesterol and high amounts of SL characterize the lysosomal lipid signature, indeed increased cholesterol levels in lysosomes are used as an early trait of numerous lysosomal storage diseases (LSDs) (reviewed in [19,95]).

Lysosomal lipids are fully obtained by lipid transport from other organelles particularly through the budding and fusion of membrane vesicles. Lysosomes form contact sites with organelles such as mitochondria, peroxisomes, and the ER. Through this contact sites Chol is transported between organelles, among other activities (reviewed in [16]). The lysosome is a hub where several trafficking pathways converge. Thus, the lysosome is a key coordinator in the sorting and delivery of lipids to several membrane compartments (reviewed in [96]). For instance, exogenous TG, sterols and PL transported by low-density lipoproteins (LDLs) first enter the cell through receptor-mediated endocytosis, and secondly, they are processed by the lysosome. Endogenous lipids are also sorted by the lysosome when it fuses with double-membraned autophagosomes. Hence, the role of lysosomes is well-established in lipid catabolism and in dietary lipid overload, when cellular lipid content or composition induces changes to alter autophagic activity in a tissue-specific manner (reviewed in [97]).

### 3.6. Nuclear Membrane

Eukaryotic nucleus encloses this chromatin using a double membrane (nuclear envelope, NE). NE includes three interconnected domains with morphological differences: the inner (INM) and outer (ONM) nuclear membranes (NM) and the pore membranes. These two individual lipid bilayers are separated by a luminal space, of 30–50 nm in human cells, named lumen or perinuclear space. Both the INM and ONM possess unique composition and metabolic/signaling patterns (reviewed in [98,99]).

Cell nucleus contains GPL, SL, and Chol. Major nuclear GPL include polyphosphoinositides (PPIn) including phosphatidylinositol phosphate (PInsP), phosphatidylinositol 4,5- bisphosphate (PtdIns(4,5)P2), and phosphatidylinositol 1,4,5- trisphosphate (PtdIns(1,4,5)P3), PtdChol, PtdSer, and PtdEtn. SL include SM, Cer, ceramide-1-phosphate (C1P), Sph, S1P and gangliosides as well as Chol and its hydroxyl and oxygenated derivatives. Total GPL contents of nuclei are reported as 3% by weight compared with 75% for protein and 22% for DNA. Cellular nuclei contain high levels of PtdCho and SM, which are partially linked with Chol and proteins to form lipid–protein complexes. The most abundant nuclear SL is SM and is the most prominent member of the SL contributing to the signaling through NE [100]. Gangliosides are another SL associated with the nucleus (reviewed in [101,102]).

SM not only occurs in the nuclear envelope, but also at intranuclear sites. Nuclear lipids exert different functions upon their localization, including the regulation of fluidity of both NM and nuclear matrix. Another function of nuclear lipids is serving as platforms in signal transduction for vitamin and hormone receptors and for anchoring active chromatin. Furthermore, nuclear lipids play a role in DNA duplication and the regulation of gene expression and transcription (reviewed in [102]). The nucleus further hosts the metabolizing enzymes for gangliosides and Cer, including sphingomyelin synthase (SMS) and sphingomyelinase (SMase), ceramide kinase (CerK) and sphingosine kinase (SphK) activities (reviewed in [101]). In short, the nuclear envelope contains the bulk of nuclear lipids, while the nuclear matrix contains enzymes and metabolites necessary for autonomous lipid metabolism in the nucleus [103].

#### 3.6.1. Nuclear Size

During NE reassembly after cell division, cell needs to adjust the proper nuclear size. With this aim, cells adjust the portion of ER membrane integrated into the NE (first integrated into the ONM and then integrated into the INM). A possible mechanism to adjust this integration is limiting the movement of PL from the ONM to the INM by using nuclear pore complexes (NPCs) as a steric barrier profiting from their location at the intersection of both membranes. Another possible mechanism to modulate nuclear size also involves NPCs, those structures affect PL metabolism by constraining the subcellular position of the effector enzymes. This is the case of lipin and numerous other lipid-generating proteins recognized in the NE proteome (reviewed in [104]).

#### 3.6.2. Nuclear Phospholipid Regulation of Chromatin

Lipid–nucleic acid interactions have been hypothesized to be the basis for the organization and expression of cellular genome (reviewed in [105]). Around 50 years ago, a role of bacterial membrane was hypothesized in the segregation of bacterial chromosomes [106]. This was the seed of subsequent studies on DNA–membrane (DNA–lipid) interactions, mostly in the 1970s and 1980s, and led to the isolation of DNA–membrane complexes (DMC).

PL in the nucleus can be found far from the membranes linked to water-soluble proteins. PL’s presence in the nucleoplasm was reported for sterols, PL, sphingosines, and very hydrophobic FA where those molecules were implicated in transcriptional regulation (reviewed in [107]). Furthermore, lipids were reported to serve as hormone-like ligands within the nuclear receptor superfamily. This family involves ligand-regulated transcription factors exerting both the activation and repression of transcription. Identified ligands include some PL, hydrophobic, Chol-based molecules (e.g., steroids and oxy-sterols), and FA (reviewed in [108]).

## 4. Cellular Mechanisms of Physicochemical Membrane Homeostasis

The amphiphilic nature of PL leads their spontaneous distribution into bilayers in aqueous environments, which are the basic component of all cellular membranes. The nature of their head-group and the length and saturation of their acyl chains define the category of PL species and the structural features of the membrane, i.e., viscosity, curvature, and electrostatic charge of the bilayer (reviewed in [66]). The physiological particularities of subcellular organelles are based on their membrane properties, including lipid composition which indeed needs to be homeostatically preserved to maintain the organelle’s physiological purpose. Membrane protein abundance and composition also affect the physicochemical properties of biological membranes. The most robust demonstration of this influence comes from experiments where lipids isolated from biological membranes self-assemble into non-lamellar phases, not into bilayers as they do in their natural, protein-rich environment [109].

**Figure 3:**
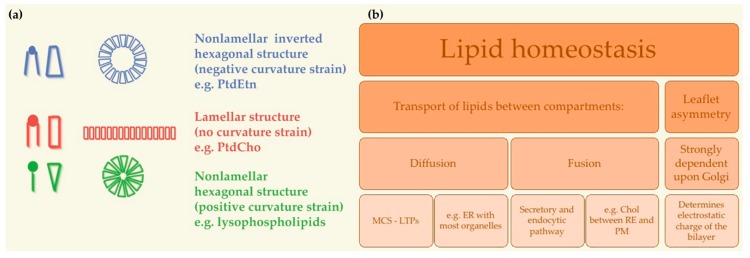
Membrane homeostasis and circuits of lipids between organelles. (**a**) Lipid bilayers can form a number of different structures upon their composition in PL species. Some examples of lipid shapes and their influence on membrane structure are shown. Lipids with a small polar head (blue), such as PtdEtn, have a molecular shape that resembles a truncated cone. They induce a negative curvature strain. Lipids with a bulky polar head and only one acyl chain (e.g., lysophospholipids, green) have a molecular shape similar to an inverted cone and induce a positive curvature strain in membranes. Lipids such as PtdCho have similar cross-sectional areas for the polar head and hydrophobic region and resemble cylinders (red). They form lamellar phases, with no curvature strain. Not to scale. Adapted from [113]. (**b**) Mechanisms of lipid homeostasis maintenance between cell compartments. Chol: cholesterol; ER: endoplasmic reticulum; MCS: membrane contact sites; LTPs: lipid transfer proteins; PM: plasma membrane; PtdCho: phosphatidylcholine; PtdEtn: phosphatidylethanolamine.

Further focusing on PL, PtdCho amount is beyond 50% of all PL in most eukaryotes. PtdCho commonly involves one cis-unsaturated acyl chain, for instance oleic acid (C18:1). This cis-double bond makes a strong kink that undoubtedly decreases the packing compactness of the acyl chains and in turn rises membrane fluidity [110]. PtdCho owes to its cylindrical shape the capability to self-assemble spontaneously (Figure 3). Hence, at a physiological temperature, PtdCho arranges into closed bilayers in a liquid crystalline state. All in all, PtdCho is specially endowed to keep a stable and fluid ground for cellular membranes (reviewed in [17]). By contrast, the conical shape of PtdEtn, due to the relatively small polar head group, imposes negative curvature stress on the membrane. Acyl chain unsaturation in PtdEtn further increases the non-bilayer propensity of this PL, creating lipid-packing defects that facilitate membrane fusion and influence the binding and activity of peripheral membrane proteins [111,112]. Though in comparatively low abundance, PtdSer and PtdIns are crucial determinants of membrane surface charge and, further on, mediate operational connections with positively charged portions of membrane proteins, both peripheral and integral [40,41] (reviewed in [17]).

As previously introduced, at least some lipids in every organelle were synthesized elsewhere and gained through transportation. Lipid transport between organelles needs to be specific in order to preserve the exclusive lipid composition of the organelles involved in the transfer (reviewed in [16]). 

The plasma membrane, endosomes and lysosomes depend completely on lipid transport from other organelles (reviewed in [16]). Lipid transport can occur by several mechanisms. The first mechanism is diffusion: lipids can laterally diffuse through membrane continuities, such as those that exist between the ER and the ONM and INM. Membrane contact sites (MCS) with ER were reported for almost every other organelle. MCS are defined as confluence points where the membranes of two organelles are positioned close to each other so as to offer a milieu where proteins in one membrane are accessible to the opposite membrane. Indeed, MCS are the purpose-built platforms for the allocation of distinct classes of lipids from the ER to other organelles using non-vesicular transport. This transfer is mediated by lipid transfer proteins (LTPs), which, in MCS, can easily reach the lipid molecules to be transferred between organelles (reviewed in [114]). Tubular connections have been observed between Golgi cisternae. On the contrary, mitochondria and peroxisomes do not take part into the vesicle transport system and lipids are probably coming in and out of these organelles as monomers, aided by both water-soluble and membrane-based proteins (revised in [16]). The second mechanism is fusion: the major membrane transport pathway between cellular organelles in the secretory and endocytic pathways is through the budding and fusion of membrane vesicles. Particularly remarkable is the transport of Chol between ER and the PM. The PM concentrates 80% of total cellular cholesterol but lacks sterol regulatory element-binding proteins (SREBPs). In contrast, the ER membrane, with less than 1% of total cellular cholesterol, contains both SREBPs and the sensors controlling SREBP activation [115] (reviewed in [116]). Careful coordination of the lipid transport between PM and ER enables ER cholesterol sensors to continuously follow PM cholesterol content [117]. Control of the carrying of cholesterol from the PM to the ER is governed by the arrangement of cholesterol in PM, which in turn is crucially restrained by the connection of cholesterol with SM [118] (reviewed in [116]). 

The electrostatic charge of the bilayer is another element responsible for the maintenance of an organelle’s physiological identity and function. Asymmetric distribution of PLs between both leaflets is strongly dependent upon the Golgi apparatus. In the Golgi, the precise asymmetric distribution of PLs between both leaflets of the PM is established, and therefore the corresponding effect on membrane electrostatics. On one hand, PtdSer fluctuates from a higher concentration in the luminal leaflet of the ER to an opposite distribution at the PM, where PtdSer faces the cytoplasm and contributes to the negative surface charge [16,119]. Type 4 P-type ATPases (P4-ATPases) in the trans-Golgi are the responsible for translocating PtdSer and other PL from the luminal to the cytoplasmic leaflet [120,121]. On the other hand, PtdIns restriction to highly dynamic sub-compartments of the ER further suggests that cytoplasmic surface of ER is overall relatively neutral [43]. 

As a reflection of physicochemical membrane homeostasis, the lipidome of cellular organelles is remodeled, and it does much faster than the proteome [122,123,124,125]. Two examples of physiological processes modulating the lipidome are metabolic challenge [124] and acute stress [125]. A third example is cellular differentiation [122,123]: dramatic lipid composition remodeling was reported in early post-natal differentiation of synaptic membranes from mammalian neurons [122]. Against this lipidome flexibility, the fluidity and packaging of the membranes remained intact in this neural model, suggesting that physical homeostasis prevails over changes in lipid composition [122]. A very tempting prospect might be that instead of preserving membrane lipid compositions, cells pursue the retention of their attributes by adjusting the multifactorial lipidome (reviewed in [15]).

## 5. Lipid Imbalances and Human Pathologies

**Figure 4:**
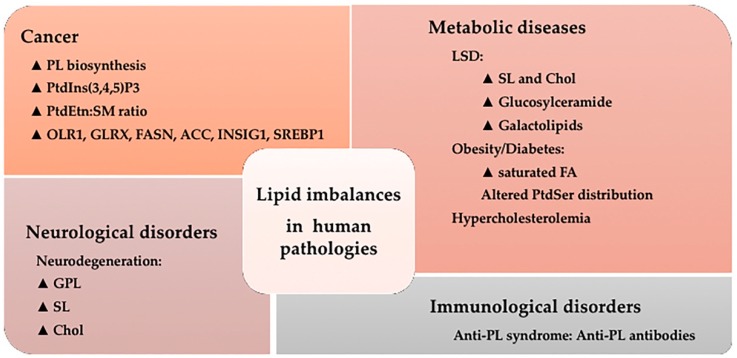
Lipid imbalances and human pathologies. Snapshot of the alterations in the lipidome which have been described in a variety of pathological situations. ▲, increased levels or pathway activity; PL: phospholipid; PtdIns(3,4,5)P3: phosphatidylinositol 3,4,5-trisphosphate; PtdEtn: phosphatidylethanolamine; SM: sphingomyelin; OLR1: Oxidized Low Density Lipoprotein Receptor 1; GLRX: Glutaredoxin; FASN: FA Synthase; ACC: acetyl-CoA carboxylase; INSIG1: Insulin induced gene 1; SREBP1: sterol regulatory element-binding protein 1; LSD: Lysosomal disorder; SL: sphingolipid; Chol: cholesterol; FA: fatty acid; PtdSer: phosphatidylserine; GPL: Glycerophospholipids.

The lipidome of cellular organelles is remodeled not only upon physiological conditions but also under pathological conditions (Figure 4). Those changes in the cell membrane lipid composition, in turn, affect the biophysical properties of microdomains and the ability of signal propagation by proteins and signaling lipids (reviewed in [19]). For instance, several different pathologies develop with alterations in lipid raft composition and structure [126,127,128]. With further detail, PL regulate endocytic processes which are required for properly regulated growth, immune surveillance, synaptic transmission, cell migration, and many other functions. Hence, alterations in PL metabolism during endocytosis were reported in chronic infections, metastatic tumors, and neurological disorders (reviewed in [66]). A further example involves human disorders, such as Alzheimer’s disease and type II diabetes, which develop with accumulations of peptide and protein fibrillar aggregates. In those cases, oligomer-mediated cytotoxicity does not depend simply on the protein aggregates themselves, but also on the chemical composition and physicochemical features of the cell membranes with which the oligomers interact [129,130,131,132].

Cystic fibrosis causes lipid imbalances which impair surfactant ability in the lungs and promote bacterial growth, leading to decreased breathing ability [133,134].

### 5.1. Cancer

Cancer cells require significant cellular growth and therefore membrane biosynthesis to survive. In accordance, PL biosynthetic pathways are up-regulated in cancer [102,135]. Several alterations in membrane composition and PL synthesis, distribution, or catabolism reported in cancer are listed below:

- PtdIns(3,4,5)P3 concentration is also elevated in numerous cancers. This example of abnormal lipid catabolism is due to ineffective degradation by the tumor suppressor phosphatase and tensin homolog (PTEN) [136,137].
- Total SM levels are decreased and PtdEtn levels are increased in tumor cells compared to non-tumor controls [138]. As a consequence, the PtdEtn:SM ratio in tumor cells is ca. 10-fold the ratio in normal cells. In fact, PtdEtn:SM ratio might work as a switch where high PtdEtn:SM ratio is a ‘ON’ state allowing propagation of proliferation signals received at the PM, while a low PtdEtn:SM ratio is a ‘OFF’ state where the PM is impaired for transduction of proliferative signals [139].
- Other SL involved in cancer pathology include Cer, Sph and S1P (reviewed in [140]). Cer mediates numerous cell-stress responses, such as induction of apoptosis [141] and cell senescence [142], whereas S1P in contrast exerts its role in cell survival, migration, and inflammation [143]. Further investigations point to an abnormal SL signaling in carcinogenesis of various types of cancer due to alterations in the activity of enzymes that participate in the metabolism of SL [144].
- Other lipid metabolism genes related with cancer are Oxidized Low Density Lipoprotein Receptor 1 (OLR1) and Glutaredoxin (GLRX), which are upregulated in breast and prostate cancer tissues [145]. The oncogenic antigen-519, a molecular marker found in breast cancer patients with poor prognosis, was identified as FA Synthase (FASN) already twenty-five years ago [146]. Other proteins related to FA biosynthesis and lipid metabolism regulation, such as acetyl-CoA carboxylase (ACC), Insulin induced gene 1 (INSIG1), and sterol regulatory element-binding protein 1 (SREBP1), are highly expressed in breast cancer tumors and associated with low patient survival [147] while colorectal carcinoma risk has been associated with hepatic lipase polymorphisms [148]. Alterations in lysosomal SL metabolism are another trait of many cancers [149].

The above-mentioned lipid traits of tumors reflect their important roles during tumor initiation and disease progression at different points (reviewed in [135]):

▪ Disruption of normal tissue architecture: The collapse of normal tissue architecture is a mark of malignancy. Polarized epithelia turn into disorganized structures that can occupy the adjacent tissues. In epithelial tissues, aberrant FA synthesis was linked to the loss of cell polarity. Also, the ordinary expression of SREBPs is needed to maintain the apical surface of normal epithelial cells and is lost in many cancers (reviewed in [135])
▪ Cancer cell migration: Cell migration stimulation by pro-migratory signaling lipids, such as DAG, LPA and prostaglandins, has been well characterized (reviewed in [150]). In addition, other findings suggest that dietary-derived lipids might enlarge the overall lipid composition of malignant cells and so influence multiple signaling events within tumors [151].
▪ Interaction of cancer cells with components of the tumor stroma: This is another key element affecting tumor growth [152] and lipids were also involved in this communication. Examples include the support to cancer-associated fibroblasts by the expression of FASN [153], the compromise of proper macrophage functioning upon FA biosynthesis [134,154] and of proper immune response upon prostaglandin presence [155].
▪ Lipid metabolic reprogramming in cancer cells: cancer cells display an enlarged metabolic inventory that allows the flexibility to survive and grow in the severe tumor environment. Highly proliferative cancer cells display a strong lipid and Chol avidity, which they fulfill by raising the incorporation of exogenous (or dietary) lipids or increasing their endogenous synthesis. Excessive lipids and cholesterol in cancer cells stored in lipid droplets are considered marks of cancer aggressiveness (reviewed in [156]).

### 5.2. Metabolic Diseases

Several lysosomal storage disorders (LSDs) affect lysosomal hydrolases, membrane transporters, accessory or trafficking proteins and derive into lipid storage disorders (sphingolipidoses, gangliosidoses, leukodystrophies) (reviewed in [95]). The most common LSD is Gaucher disease (GD), which impairs the function of (lyso)glucosylceramide degrading enzyme β-glucocerebrosidase (EC 3.2.1.45). Fabry disease (FD), in contrast, develops with deficiency of the lysosomal α-galactosidase A (EC 3.2.1.22). Krabbe disease (KD) interferes with the function of the enzyme galactocerebrosidase (EC: 3.2.1.46), so that undegraded galactolipids accumulate and cause the progressive demyelination of cells in the nervous system. Niemann–Pick type C disease originates by intralysosomal Chol and SL accumulation and develops with severe neurological and visceral pathology (reviewed in [95]). Another example is Tay Sachs disease, a rare inherited disorder that progressively destroys nerve cells (neurons) in the brain and spinal cord. This disease is a type of sphingolipidosis which shows toxic accumulation of the molecule GM2 ganglioside within cells [157].

Obesity is another metabolic disease initiated by a lipid imbalance. Particularly, it has been linked to the intake of saturated (or trans-monounsaturated fatty acid –MUFA) fats (reviewed in [19]). One of these saturated FA, palmitic acid, has been shown to induce of ER stress and cell death [158].

Diabetes is a disease highly influenced by the dietary FA content. In diabetic patients, increased saturated:unsaturated FA ratio is found in erythrocyte membranes compared to healthy controls [159]. In addition, dysregulation of PtdSer asymmetric distribution in the PM may contribute to the development of diabetes [160,161].

Focusing on the molecular mechanism behind the onset of lipid imbalance-driven obesity and diabetes, both lipid homeostasis and physical principles governing lipid arrangements in the membranes are involved. Two examples illustrate this mechanism in liver disease (reviewed in [17]). The liver is a crucial metabolic organ in controlling body energy metabolism. It functions as a metabolic hub linking several tissues, skeletal muscle and adipose tissue included. In fat metabolism, the liver cells break down fats and produce energy (reviewed in [162]).

▪ In the first example, excessive intake of saturated FA is highly toxic for hepatocytes due to its limited capacity to integrate them into TG [163]. In parallel, an elevated degree of membrane saturation disrupts calcium homeostasis and triggers liver ER stress [164]. Chronic activation of ER stress dysregulates lipid homeostasis and might lead to dyslipidemia, insulin resistance, type II diabetes, and obesity [163].
▪ A second example involves the previously introduced TFEB, transcription factor partially dictating lysosome biogenesis. As above mentioned, TFEB participates in autophagy and in the clearance of lipid droplets. Furthermore, it governs liver lipid catabolism and energy metabolism interfering with peroxisome proliferator-activated receptor-γ (PPAR-γ) signaling [165]. In obese animals, TFEB overexpression rescues obesity and associated metabolic syndrome by promoting lipophagy [166].

A high fat diet can likewise obstruct the formation of lipid rafts needed for the uptake of exogenous factors such as insulin from the blood [167,168].

Hypercholesterolemia disease shows defective clearance of low-density lipoproteins (LDL) due to mutations in elements of the sterol and lipoprotein pathways. Furthermore, elevated plasma Chol levels associate with higher levels of Chol in the PM of cardiovascular and other cells, and in turn alter PM structure and signal transduction (reviewed in [169]).

### 5.3. Neurological Disorders

The neural membrane lipid composition has been reported to change in neurodegeneration. Particularly, changes affected GPL, SL, and Chol levels both in acute neural trauma (head injury, ischemia, and spinal cord trauma) and in neurodegenerative diseases (Alzheimer’s, Parkinson’s and amyotrophic lateral sclerosis). In accordance, significant increased levels of in GPL-, SL-, and Chol-derived lipid mediators have been shown to accompany these variations in lipid composition both in the PM and in the cell nucleus (reviewed in [101]).

### 5.4. Immunological Disorders

Anti-phospholipid syndrome (APS) is an autoimmune disease caused by the appearance of anti-PL antibodies and developed with vascular thromboembolism and miscarriages (reviewed in [170]). Anti-PL involve lupus anticoagulant (LA), anti-β2GPI antibodies and anti-cardiolipin (anti-CL). The mechanism linking the binding of anti-PL antibodies to endothelial cells with cell activation is still under investigation. A great variety of infections are accompanied by increases in anti-PL mostly in viral infections (Hepatitis C virus, Epstein-Barr virus, Varicella virus, human immunodeficiency virus) (reviewed in [171]).

The avoidance of autoreactivity to the broadly distributed self-PL is a key instrument to prevent autoimmune diseases. However, little is known on the molecular mechanisms responsible of T cell autoreactivity on lipid antigens (reviewed in [172]).

### 5.5. Regulation of Cellular Activities by Prenylation

Protein prenylation is a ubiquitous covalent post-translational modification. It is the first critical step for membrane targeting and binding and mediates protein-protein interactions [173]. This modification increases the hydrophobicity of the protein [174], favors its temporary attachment to the membrane [175] and is essential for a correct protein activity [173]. Protein prenylation consists of the attachment of farnesyl (15 carbon) or geranylgeranyl (20 carbon) groups to the protein [176]. First described in fungi [177], farnesylation was described in mammals on lamin B [178,179]. Defective prenylation of proteins is linked to tumor pathogenesis among others [180].

Three enzymes catalyze the prenylation of proteins: farnesyltransferase (FTase) and geranylgeranyltransferases type I and II (GGTase I and GGTase II). They are heterodimers recognising C-terminal consensus sequences in the target proteins. FTase and GGTase I transfer a single farnesyl or geranylgeranyl group to a cysteine residue located in the “CaaX box” (C-terminal consensus sequence in which “C” represents a cysteine, “a” is an aliphatic amino acid and “X” determines the attached isoprenoid) [176]. Proteins modified by FTase or GGTase I need two additional processing steps: cleavage of “aaX” tripeptide by an endoprotease and methylation of prenylcysteine [174]. The structure and enzymology of FTase and GGTase I are similar, although most studies focus on farnesylation. First, farnesyl residue binds to FTase [181,182], secondly the protein with a CaaX-box binds and the C-S bond is formed. While the prenylated protein remains bound to the enzyme, another prenyl group binds to the enzyme and, lastly, the product (prenylated protein) is dissociated before or while a new CaaX-box substrate binds [173]. As a consequence, stereochemistry at C-1 position of the isoprenoid is inverted [183,184,185] but this causes minimal differences in protein conformation [186]. FTase and GGTase I have not mutually exclusive substrate specificity [187], although there are preferences depending on the “X” aminoacid in CaaX box, as said above, so some prediction tools have been developed.

GGTase II, also called Rab GGTase, catalyzes the transference of two geranylgeranyl groups to two cysteine residues located in sequences such as CXC or CCXX close to C-terminus of Rab proteins [176]. Its enzymology is more complex and needs an escort protein to catalyze the reaction [188].

Although, only a few prenyltransferase substrates (e.g., Ras family GTPases and heterotrimeric G-proteins) have been identified, this modification is essential for the proper activity of some proteins which are altered in different diseases. This is the basis for the efforts made in the therapeutic targeting of protein prenylation. Inhibitors of prenylation have been tested for the treatment of cancer, parasite causing diseases, viral and bacterial infections, multiple sclerosis or progeria [173].

Proteins involved in signal transduction pathways require prenylation. An interesting example of these proteins is Ras protein family. Many cancers present activating mutations in the RAS genes causing malignant activity of the protein [189]. For this reason, prenyltransferase inhibitors have been tested in vitro and in animals for the inhibition of Ras proteins, showing promising results. Firstly, farnesyltransferase inhibitors (FTIs) were used to inhibit the farnesylation of proteins in cancer cells. FTIs promoted apoptosis, cell cycle arrest (mainly mitotic arrest at prometaphase) and inhibition of proliferation, cell migration and angiogenesis by affecting signal transduction pathways such as RAF-MEK-MAPK or PI3K-Akt and other oncogenic and survival pathways [180]. Unfortunately, clinical studies did not show robust anticancer activity [190] with only several patients responding to FTIs both alone and in combination with other agents. In sum, some tumors depend on farnesylated proteins for survival, but further development is needed to apply this knowledge to the clinic [180].

More recently, geranylgeranyltranseferase inhibitors (GGTIs) were developed. Ras protein isoforms were found to be geranylgeranylated when FTase was inhibited and some pathways downstream Ras were mediated by geranylgeranylated proteins [191]. In cell and animal models, GGTIs block cell cycle in the G1 phase [192], induce apoptosis, and inhibit tumor cell growth [180].

Other protein susceptible to be targeted by prenylation inhibitors include proteins being exclusively farnesylated (e.g., HRAS and RAS homologue enriched in brain –RHEB) or geranylgeranylated (e.g., RHOA, RHOC, RALA and RALB); although some can be both farnesylated or geranylgeranylated (e.g., RHOB) or become geranylgeranylated when farnesylation is inhibited (e.g., KRAS, as said above, and HRAS). Other prenylation inhibitors’ targets include tumor suppressors (such as ARHI, RERG, DBC2, RIG, RRP22) and other proteins (such as CENPE, CENPF, PRL phosphatases, lamin A and B, HDJ2, RND3, PEX19, RHOD, RHO6, RHO7, TC10 and PTGIR) (reviewed in [180]).

## 6. New Lipid-Based Therapeutic Avenues: Membrane Lipid Therapy

### 6.1. Molecular Bases of Targeting the Plasma Membrane

In the above sections it was made clear that many cell functions take place in membranes and their surroundings [160]. Furthermore, it was reviewed how membrane components participate in functions altered in many human diseases [19]. Those functions include changing the localization of key proteins in the cell, in turn, altering key protein-protein interactions in membrane microdomains. Subsequently, signaling cascades are altered and correct functioning of the cell is compromised. Hence, the new therapeutic approach, membrane lipid therapy (MLT), aims to target membrane components to modify membrane composition or structure, i.e., the drug-induced regulation of membrane lipid composition and structure [113]. This strategy has shown not only efficacy, but also higher specificity and safety, and has become a potential alternative to conventional drugs [6].

The variety of membrane microdomains based on different compositions and bearing specific biological properties was already introduced [45]. Furthermore, membrane microdomain composition, and subsequently function, may be modified by both lipid intake and enzymatic regulation [19]. Proteins involved in cell signaling interact with these microdomains and assemble crucial signaling platforms whose alteration might be pathological [126,127]. Thus, MLT aims to alter this protein signaling by modifying the composition and structure of the membrane microdomains and subsequently modulate cell signaling, offering potentially effective treatments for a variety of conditions [113,193,194,195]. The overall membrane lipid composition, the composition of membrane microdomains or the composition of internal organelles can be targeted by natural or modified lipids or drugs [196,197,198]. For instance, proteins such as G proteins or protein kinase C (PKC), as well as stress response proteins or enzymes such as SMS alter their binding to membrane according to membrane composition and structure [19].

**Figure 5:**
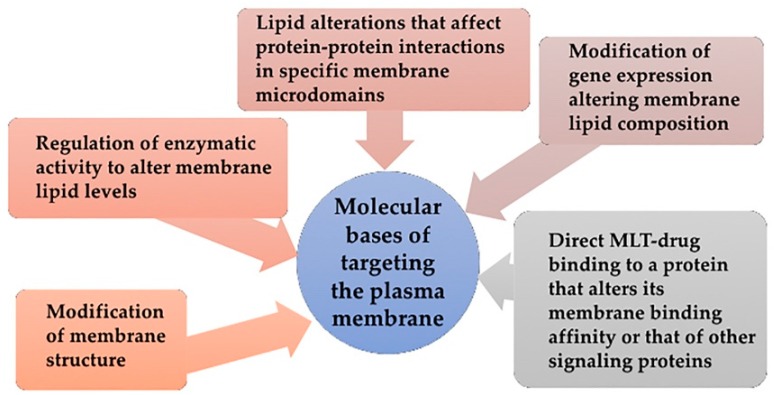
Molecular bases of Membrane-Lipid Therapy. Snapshot of the rationale behind targeting membrane composition or structure i.e., the drug-induced regulation of membrane lipid composition and structure.

Different regulatory effects might be obtained when targeting plasma membrane for therapy (Figure 5):

- Direct regulation through membrane structure modification: Dietary lipids and environmental changes modify cell membranes changing their properties and microdomain organization, thus controlling the localization and activity of proteins such as G proteins (interaction with membrane and downstream signaling) [199,200], the transcription of proteins involved in stress response such as heat shock proteins (Hsp) [201], or the production of second messengers such as Cer [202]. Thus, MUFA treatments can change the order of Lo and Ld microdomains.
- Regulation of enzymatic activity to alter membrane lipid levels: Enzymatic activity of SMS and other enzymes from SL metabolism are altered in cancer and consequently modify membrane composition and structure. Hydroxyl-C18 unsaturated fatty acids (hydroxyoleic, hydroxilinoleic, hydroxy-α-linolenic and hydroxy-γ-linolenic acids) have effects on SMS activity [138].
- Modification of gene expression that results in alterations of membrane lipid composition: this gene expression change might affect the activity of an enzyme, protein-lipid interactions or protein-protein interaction. DNA-associated PL are found in nuclear membrane [203] and their effects on nuclear functions have been documented [103]. Evidence of this type of MLT include the increased SM levels in differentiated cells in contrast to low levels of this lipid on proliferating cells [138]. This increase does not take place with SM addition but for SMS increased activity [138,139], which supports the concept of MLT-induced gene expression alteration.
- Lipid alterations that affect protein-protein interactions in specific membrane microdomains: The alteration of lipid ratios or the presence of particular lipids in membranes cause changes in protein-protein interactions. For example, low PtdEtn/SM ratios reduce Ras interactions both with membrane components and with downstream partners, in turn, inhibiting the transduction through proliferative signaling cascades and preventing proliferation in cancer cells [139].
- Direct MLT-drug binding to a protein that alters its membrane binding affinity or that of other signaling proteins: In that case, the MLT formulated molecule binds to a protein rather than a lipid. This is the case of prenylation inhibitors, which prevent Ras from binding to membrane and subsequently inhibit cancer cell proliferation while inducing cell differentiation and cell death [139].

### 6.2. Development of Membrane Lipid Therapy in Different Therapeutic Areas

**Figure 6:**
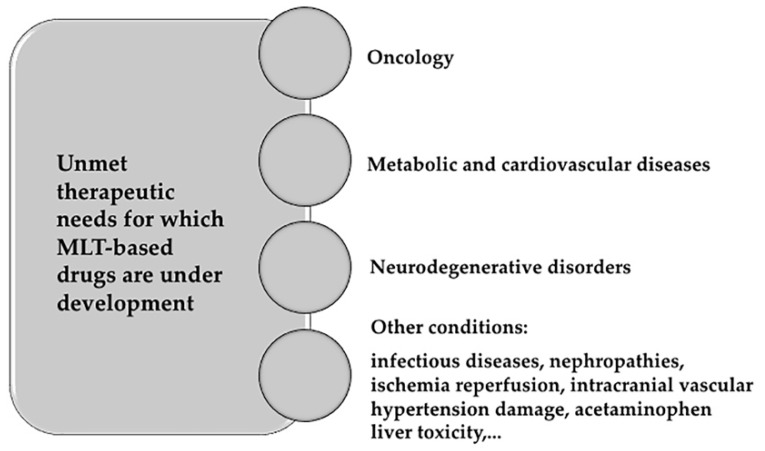
Membrane-Lipid Therapy molecules are currently being developed for several medical conditions.

In MLT, synthetic fatty acids are newly designed with the aim of regulating membrane microdomain distribution mimicking natural lipids [204]. Thus, MLT is a sophisticated therapeutic approach to make protein–protein interactions targetable in an environment where those interactions are not amenable to manipulation [139,205,206]. Many diseases are already being targeted by MLT. The most relevant fields in which MLT molecules are being developed are listed below and further reviewed in [19] (Figure 6).

- Oncology. The basis of developing MLT in oncology comes from the finding that membrane lipid composition might work as a switch allowing or compromising propagation of proliferation signals received at the PM in tumor cells [196,207]. Many molecules have been developed since the discovery membrane-altering mechanism of action of doxorubicin [199]. One of the most promising molecules is rationally-designed 2-OHOA (2-hydroxyoleic acid) [208], which is currently being tested in clinical trials for the treatment of glioma. 2-OHOA activates SMS increasing its product, SM, and decreasing its substrate PtdEtn in membranes of cancer cells but not of healthy cells because of the higher levels of SM found in tumor cells [138]. 2OHOA was the first rationally designed MLT molecule to arrive clinical trials. It has shown good pharmaceutical efficacy and safety against cancer in humans (ClinicalTrials.gov identifier #NCT01792310). After a first-in-man phase I/IIA trial in patients with solid tumors, 43% glioma patients responded to treatment, although this percentage almost doubled (ca. 80%) if patients previously pretreated with avastin were disregarded [6]. Other molecules being explored in oncology follow here: 2-hydroxylinoleic acid is on phase II clinical trial. This PUFA binds to membrane and inhibits the Akt/mTORC1 axis and induces specific cancer cell autophagy [209]. Hydroxytriolein is a triacylglycerol mimetic synthetic lipid analogue of triolein shown to block cancer cell growth in vitro through the β-catenin pathway, downregulation of the MEK-ERK axis, and production of Reactive Oxygen Species and apoptosis [210]. Worth mentioning are propofol-docosahexaenoic acid (P-DHA) and its analogue edelfosine (reviewed in [6]). Examples of anticancer therapies based on inhibitors targeting enzyme regulators of lipid metabolism are orlistat (Roche Xenical^®^), a FASN inhibitor administered in the treatment of breast cancer [211]; and ABC294640, a sphingosine kinase 2 and dihydroceramide desaturase inhibitor currently under an Ib/II clinical trial (ClinicalTrials.gov identifier #NCT02757326).
- Metabolic and cardiovascular diseases: The use of dietary lipids for the treatment of diabetes or obesity is a clear case of MLT. For example, body weight reductions where achieved in rats with daily supplements of olive oil (made of 70%–80% oleic acid) but not with supplements of its trans analogue elaidic acid, due to the different structure of both FA [44,212]. Furthermore, oleic acid analogues induce reduction of body weight in rats by promoting overexpression of uncoupling proteins UCP1 and UCP3 and decreasing food intake [212]. A high intake of oleic acid has also been shown to improve glycemic status and reduce saturated FA levels of diabetic patients while increasing those of unsaturated FA [159]. In the case of cardiovascular conditions, high oleic acid intake and ω-3 FA consumption were linked to reduced blood pressure values [213,214]. This reduction is even greater with 2-hydroxioleic acid treatment [215,216]. Furthermore, unsaturated FA are cardioprotective [217].
- Neurodegenerative disorders: Behind adipose tissue, the central nervous system concentrates the largest depot of lipids in the body making it a primary target for MLT strategies. Alzheimer’s disease risk has been inversely associated with ω-3 polyunsaturated fatty acid (PUFA) consumption [218]. In detail, the altered amyloid precursor protein (APP) proteolysis upon DHA abundance was the basis to design 2-hydroxioleic-DHA (2-OHDHA). A four-month treatment with 2-OHDHA in a severe Alzheimer’s disease mice model (5XFAD) restored cognition to control values [219,220]. Spinal cord injury might also benefit from MLT approaches (reviewed in [19]). Albumin-oleic acid complex induces significant motor recovery (~ 40%) in rats with spinal cord injury (SCI) [221], ameliorating both spasticity and pain. The oleic acid analogue NFX88 (Neurofix) is undergoing clinical trials for the treatment of neuropathic pain in patients with SCI. Finally, a phase II/III clinical trial (ClinicalTrials.gov identifier #NCT00706147) is being developed to evaluate the efficacy of the lipid interacting hydroxylamine derivative arimoclomol in familial amyotrophic lateral sclerosis.
- Other conditions that are bound to be ameliorated using MLT include infectious diseases, chemotherapeutic neuropathy, wound healing, retinopathies, nephropathies, acetaminophen liver toxicity, sunburn, ischemia reperfusion, intracranial hemorrhage, atrial fibrillation, vascular hypertension damage, and myocardial infarction as reviewed in [19].

## 7. Future Directions and Conclusions

Initially described as pure barriers, biological membranes are currently viewed as active players in cell and organelle architecture and physiology. Lipid composition peculiarities of the different cell compartments is behind their specific structural properties and functionalities. Indeed, lipid dysregulation is a relevant factor in the etiology of many diseases. To exploit these new therapeutic avenues, lipids (both natural and synthetic) are being developed as potential drugs for different conditions using the MLT approach and are bound to constitute alternative or innovative therapies for fields in which there are no available or promising treatments.

## Abbreviations

2-OHDHA	2-hydroxydocosahexanoic acid
2-OHOA	2-hydroxyoleic acid
ACC	acetyl-CoA carboxylase
ALPS	amphipathic lipid packing sensor
APP	amyloid precursor protein
APS	anti-phospholipid syndrome
BmP	bis(monoacylglycero)phosphate
C1P	ceramide-1-phosphate
CLIC	clathrin-independent carrier
CDP-DAG	cytidine diphosphate diacylglycerol
Cer	ceramide
CerK	ceramide kinase
Chol	cholesterol
CL	cardiolipin
COP	coat protein complex
DAG	diacylglycerol
DMC	DNA–membrane complexes
ER	endoplasmic reticulum
ESCRT	endosomal sorting complexes required for transport
FASN	fatty acid synthase
FD	Fabry disease
FTase	farnesyltransferase
FTI	farnesyltransferase inhibitor
GalCer	galactosylceramide
GD	Gaucher disease
GEEC	(GptdIns)-anchor-enriched endocytic compartment
GEF	guanine nucleotide exchange factors
GGTase	geranylgeranyltransferase
GGTI	geranylgeranyltransferase inhibitor
GlcCer	glucosylceramide
GLRX	glutaredoxin
GPL	glycerophospholipids
GptdIns	glycosylphosphatidylinositol
GSL	glycosphingolipid
GTPase	guanosine-5′-triphosphate hydrolase
Hsp	heat shock protein
IMM	inner mitochondrial membrane
IMS	intermembrane space
INM	inner nuclear membrane
INSIG1	insulin induced gene 1
KD	Krabbe disease
LA	lupus anticoagulant
LacCer	lactosylceramide
Ld	Liquid-disordered
LDL	low-density lipoprotein
LECA	last eukaryotic common ancestor
Lo	Liquid-ordered
LPA	lysoPA
LPC	lysoPtdCho
LSD	lysosomal storage disease
LTP	lipid transfer protein
LUCA	last universal common ancestor
MAM	mitochondria associated membrane
MCS	membrane contact site
MLT	membrane lipid therapy
MUFA	monounsaturated fatty acid
NE	nuclear envelope
NM	nuclear membrane
NPC	nuclear pore complex
NSF	N-ethylmaleimide-sensitive factor
OLR1	oxidized low density lipoprotein receptor 1
OMM	outer mitochondrial membrane
ONM	outer nuclear membrane
P4-ATPase	type 4 P-type ATPases
P-DHA	propofol-docosahexaenoic acid
PA	phosphatidic acid
PKC	protein kinase C
PL	phospholipids
PM	plasma membrane
PPAR-γ	peroxisome proliferator-activated receptor-γ
PPIn	polyphosphoinositides
PtdCho	phosphatidylcholine
PtdEtn	phosphatidylethanolamine
PtdGro	phosphatidylglycerol
PtdIns	phosphatidylinositol
PtdIns(1,4,5)P3	phosphatidylinositol 1,4,5- trisphosphate
PtdIns(3,4,5)P3	phosphatidylinositol 3,4,5-trisphosphate
PtdIns(4,5)P2	phosphatidylinositol 4,5- bisphosphate
PtdIns4P	phosphatidylinositol 4-phosphate
PtdInsP	phosphatidylinositol phosphate
PtdSer	phosphatidylserine
SL	sphingolipids
SM	sphingomyelin
SMase	sphingomyelinase
SMS	sphingomyelin synthase
SNARE	soluble N-ethylmaleimide-sensitive factor attachment protein receptor
SPC	sphingosylphosphorylcholine
Sph	sphingosine
SphK	sphingosine kinase
SREBP	sterol regulatory element-binding protein
TFEB	transcription factor EB
TMDs	transmembrane domains
UCP	uncoupling protein
